# Implementing innovative technology promoting self-awareness of brain health and self-determination in obtaining a timely dementia diagnosis: protocol for a multimethods, concurrent, two-part observational study

**DOI:** 10.1136/bmjopen-2024-088182

**Published:** 2025-06-17

**Authors:** Alison M Hutchinson, Helen Macpherson, Tanya Petrovich, Rajesh Vasa, Terence W H Chong, Lidia Engel, Tanita Botha, Tracey K Bucknall, Kelly Burns, Stephanie Daly, Justine Lomas, Kon Mouzakis, Loren Mowszowski, Sharon L Naismith, Bernice Redley, Jessica Rivera Villicana, Andrew Vouliotis, Eva Yuen, Liliana Orellana

**Affiliations:** 1 School of Nursing and Midwifery, Centre for Quality and Patient Safety Research in the Institute for Health Transformation, Deakin University, 1 Gheringhap St, Geelong 3220, Victoria, Australia; 2 University Hospital, Barwon Health, Bellerine St, Geelong 3220, Victoria, Australia; 3 Institute for Physical Activity and Nutrition (IPAN), Deakin University Faculty of Health, Geelong 3220, Victoria, Australia; 4 Dementia Australia, 155 Oak Street, Parkville 3052, Victoria, Australia; 5 Deakin Applied Artificial Intelligence Initiative, Deakin University, Geelong 3220, Victoria, Australia; 6 Department of Psychiatry, The University of Melbourne, Parkville 3010, Victoria, Australia; 7 St Vincent’s Hospital Melbourne Pty Ltd, 283 Cotham Road, Kew 3101, Victoria, Australia; 8 Monash University Health Economics Group (MUHEG), School of Public Health and Preventive Medicine, Monash University, 553 St Kilda Road, Melbourne, 3004, Victoria, Australia; 9 Biostatistics Unit, Faculty of Health, Deakin University, Geelong 3220, Victoria, Australia; 10 Alfred Health, 55 Commercial Road, Melbourne 3004, Victoria, Australia; 11 Sesnus Cognition, 225 Main Road, Blackwood 5051, South Australia, Australia; 12 Healthy Brain Ageing Program, School of Psychology, Brain and Mind Centre and The Charles Perkins Centre, The University of Sydney, Sydney 2050, New South Wales, Australia; 13 Health Complaints Commissioner, 570 Bourke St Melbourne, Victoria, Australia; 14 Centre for Urban Research, RMIT University, 360 Swanston St, Melbourne 3000, Victoria, Australia; 15 Monash Health, Clayton 3168, Victoria, Australia

**Keywords:** Dementia, GERIATRIC MEDICINE, Health Education, Health Literacy, QUALITATIVE RESEARCH

## Abstract

**Abstract:**

**Introduction:**

Diagnosis in the early stages of dementia can lead to successful delay in associated cognitive decline. However, up to 76% of Australians diagnosed with dementia have already advanced beyond the early stage of disease. BrainTrack is an evidence-based mobile application (app) designed in Australia to promote brain health self-awareness, self-determination to promote help-seeking and, ultimately, a timelier dementia diagnosis. We will evaluate user experience, implementation and social return-on-investment outcomes of BrainTrack and will report dementia-related concerns, dementia literacy, knowledge, stigma and motivation for behaviour change and explore their associations with demographic characteristics.

**Methods and analysis:**

A multimethods, concurrent, two-study observational design will be used. Study 1 will evaluate BrainTrack user experience and implementation outcomes, changes in users’ dementia literacy, dementia knowledge, perceptions of dementia-related stigma and help-seeking at five time points (baseline, 1, 3, 6 and 12 months). People residing in all states and territories of Australia will be recruited to the study via the BrainTrack app. Data collection will occur online and through teleconferencing. Approximately 1000 participants will complete all five surveys. Google Analytics data will measure adoption. App usage data will identify app use patterns. A sample of continuing app users (~n=80) and those who cease app use within 6 months (~n=20) will be interviewed to obtain in-depth information about their app use and help-seeking experience. Dementia Australia Helpline data will quantify help-seeking calls triggered by BrainTrack use. In Study 1, longitudinal outcomes will be analysed using mixed models. The economic and social value of BrainTrack will be assessed using social return on investment analysis. In Study 2, general practitioners (~n=20) currently practising in Australia will participate in semi-structured interviews conducted via online teleconferencing. Interviews will elicit perceptions of the usefulness of BrainTrack for initiating and facilitating discussions with patients about cognition and dementia. Qualitative data will be analysed thematically, followed by deductive analysis guided by the Theoretical Domains Framework.

**Ethics and dissemination:**

This study has received Human Research Ethics Committee approval from Deakin University Human Research Ethics Committee (Study 1: HREC Reference Number 2022–220) and Deakin University Human Ethics Advisory Group, Faculty of Health (Study 2: Reference Number 202_2022). Informed consent will be obtained prior to participation, either verbally for interviews or online for surveys. Study findings will be published in peer-reviewed journals and communicated to key stakeholders.

**STRENGTHS AND LIMITATIONS OF THIS STUDY**

- Uses a robust multimethods concurrent two-study observational design.
- Wide range of data sources obtains both user and health professional perspectives.
- Study sample limited to English speakers capable of using a digital device.
- Outcomes of interest, app user experience and usability may influence survey drop-out and reasons for study withdrawal will be obtained to account for this.
- Response bias is possible in interviews where strong views in favour or against the app exist, so current and ceased app users will be invited for balance.

## Introduction

 Dementia is life-limiting, of high and increasing prevalence and has a major impact on the lives of those affected. Globally, it is forecasted that 75 million people will be living with dementia by 2030, with a predicted rise to 132 million by 2050.^1^ The global cost of dementia exceeded US$1 trillion in 2018.^2^ Importantly, the high prevalence of undetected dementia suggests these figures could be substantially higher^3^; recent international research confirms that up to 75% of people with dementia in the community go undetected.^4^,^6^ Worldwide, dementia is the seventh leading cause of death and one of the major causes of disability and dependency.^7^ In Australia, dementia is the second leading cause of death^8^ and the greatest cause of disability in older people.^9^ Up to 472 000 Australians, including 1 in 12 people aged ≥65 years, are estimated to be living with dementia.^10^ By 2058, more than 1 million vulnerable older Australians will be living with dementia.^11^

Systematic review evidence^12^ and best practice guidelines^13^ highlight the importance of timely dementia diagnosis. The pathway to dementia diagnosis requires the identification and recognition of symptoms of cognitive or physical impairment followed by successful engagement with a health professional such as a general practitioner (GP), neurologist, old age psychiatrist or geriatrician.^14^ Comprehensive methods for dementia diagnosis include taking a history of cognitive decline and functional impairment in daily activities, cognitive screening such as the Mini-Mental State Examination,^15^ neuropsychological and medical investigations, blood tests and/or brain imaging, a review of current medication use and consideration of differential diagnoses.^16^ To effectively delay cognitive decline, interventions need to be implemented in the early/mild stages of disease.^17^ Recent Australian research indicates, however, that at their first consultation, 76% of people diagnosed with dementia had advanced beyond the early stage, with one in five people in the moderate-to-severe stages of disease,^18^ highlighting the extent of delay in dementia diagnosis. Through timely diagnosis and management of exacerbating factors (secondary prevention), cognitive and functional decline can be delayed, and quality of life optimised for those with dementia. For individuals who do not have dementia, addressing modifiable risk factors (primary prevention) may delay disease onset.^19^ Between 40% and 48% of dementia risk is potentially modifiable^2 19^ through primary prevention measures addressing physical inactivity, obesity, hypertension, depression, smoking and diabetes,^19^ as well as hearing loss, traumatic brain injury, alcohol use, social isolation and air pollution.^2^ Emerging evidence suggests dementia risk can also be reduced by addressing diet and nutrition,^20^ cognitive training^21 22^ and sleep disturbance.^23^ Such factors may hasten dementia progression, requiring secondary prevention measures.^2^

Dementia diagnosis is highly reliant on the person or someone close to them recognising and reporting cognitive and/or functional symptoms to a health professional; typically, this does not occur in a timely way.^24^ While there is widespread recognition of the importance of timely dementia diagnosis, there are numerous barriers to achieving a formal diagnosis.^25^ Such barriers include difficulties initiating discussions with GPs about cognition concerns, GPs not recognising subtle or early cognitive change (lacking knowledge of the person’s baseline cognitive function) or attributing change to ageing,^26 27^ insufficient screening tools for early diagnosis, fragmented service systems, confusing referral processes and lack of clarity about diagnostic pathways.^25^ Despite these barriers, GPs have been identified by individuals as the first health professional consulted and the preferred source of advice when seeking to address subjective concerns about memory,^28 29^ and GPs themselves recognise the importance of preventative healthcare for conditions including dementia.^30^ Therefore, GPs are ideally positioned to initiate the diagnostic pathway. However, Australian evidence indicates 1.9 years, on average, elapse between noticing symptoms and the first health professional consultation.^14^ This delay in diagnosis is of immense concern. While not curable, timely detection of cognitive decline is critical when there is a window of opportunity to intervene with secondary prevention strategies to delay disease progression,^2^ initiate treatments, implement strategies to preserve independence and delay decline^12^ and provide support to optimise quality of life.^13 31^ Furthermore, the person’s experience of their first consultation can be influential in future communication and assessment; feeling heard by GPs had a positive influence.^26 32^ Hence, strategies to enable the person to discuss their concerns and to secure the attention of their GP to commence the diagnostic pathway are urgently needed.

Help-seeking (ie, consumer-initiated discussions with health professionals about cognitive symptoms and concerns) benefits people in the early stages of cognitive decline when more intervention options are available to prevent or delay cognitive and functional decline^33^ and the person has greater capacity for insight into their disease.^34^ Promoting self-determination in help-seeking (ie, giving the person control over when, as well as guidance on how to seek help) is key to promoting timely diagnosis.^35^ However, consumer-related barriers to help-seeking include limited knowledge of symptoms, risk factors, treatments and available services for dementia,^36^ as well as concern about lack of a cure and fear about stigma.^37^,^39^ Dementia literacy and knowledge about the benefits of timely diagnosis can motivate help-seeking and subsequent diagnosis.^36 40 41^ Education can also reduce dementia-related fear and stigma.^40 42^ Further, research indicates people are willing to monitor their own cognitive health.^26^ Digital health technologies (eg, applications, or ‘apps’) can empower individuals, offer convenient, equitable and sustainable strategies that can be used anywhere and anytime.^43^ Evidence shows that technology use is pervasive in older Australians with varying degrees of cognitive concerns (ie, healthy older adults, individuals with subjective memory concerns and those with mild cognitive impairment), who demonstrate significant interest in accessing digital interventions targeting cognition and risk factors for cognitive decline^44^; thus there is an appetite for engagement with apps among community-based older adults. Leveraging digital technologies to empower individuals can improve their physical, social and psychological well-being and increase choices about the use of health and social care services, directly reducing their subjective experience of vulnerability.^45 46^

BrainTrack (box 1) was developed by Dementia Australia, the peak advocate body for people impacted by dementia in Australia,^47^ in collaboration with researchers from Deakin University. The BrainTrack app is the result of a co-design and pilot testing process inclusive of people living with or at risk of dementia and health professionals (eg, cognition specialists and GPs). Taking a wellness approach and informed by behaviour change theory, BrainTrack uses games to monitor cognitive performance and provides messaging about brain health and risk reduction strategies, including primary prevention strategies (to prevent onset of disease),^48^ and secondary prevention strategies (to prevent severe disease through detection prior to the onset of symptoms).^49 50^ By improving dementia literacy and knowledge (known to help reduce stigma), providing links to risk prevention and reduction resources and enabling personal monitoring of cognition over time, BrainTrack is designed to facilitate self-determination in help-seeking (proactive health management). Hence, this unique app is designed to enhance the lives of a large population of vulnerable older Australians by guiding people with concerns about brain health to seek help (access the health system/GP). Health professionals may then initiate the diagnostic pathway to achieve a timely diagnosis and implement interventions to delay decline and improve the quality of life for older Australians.

Box 1 **About BrainTrack**

**Figure 1:**
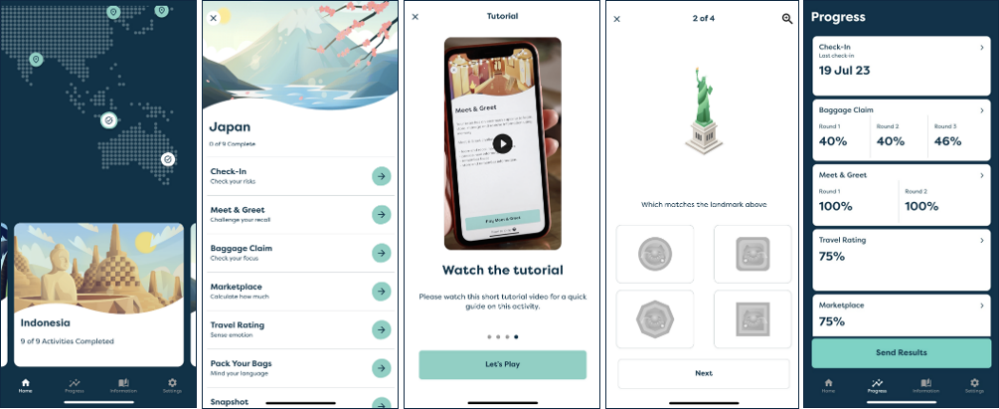
Examples of BrainTrack app features.

**Figure 2:**
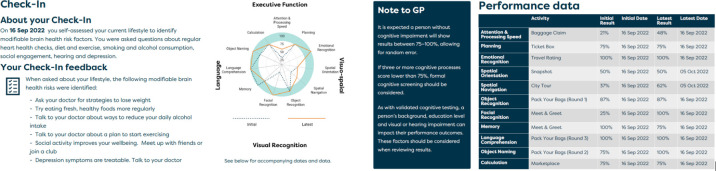
Examples of Insights Feedback Report.

Available on Android and iOS devices, BrainTrack takes users to new travel destinations every 30 days for 12 months. Each destination comprises eight games based on validated neuropsychological instruments that assess verbal ability and five cognitive domains primarily affected by dementia: memory and learning, executive function, complex attention, social cognition and visuospatial abilities. A downloadable report (Insight Feedback Report) allows users to track acute and longitudinal accuracy on each game and can be easily shared with others, including health professionals. BrainTrack also includes brain health resources, guidance on understanding and using results and contact details for the National Dementia Helpline (Australia). Figure 1 illustrates select pages from BrainTrack and figure 2 illustrates select pages from the Insight Feedback Report.

The BrainTrack app is a unique, innovative and engaging digital technology that is freely available to monitor brain health, promote brain health self-awareness and foster self-determination in help-seeking about subjective cognitive concerns. In this research, a structured, rigorous approach is used to investigate the user experience, implementation outcomes (eg, help-seeking, uptake) and the social return-on-investment (SROI) of BrainTrack. The potential of BrainTrack to reduce dementia-related stigma, promote positive health and lifestyle behaviours and promote dementia literacy, knowledge and help-seeking about brain health concerns to ultimately increase the potential for obtaining a timely dementia diagnosis will also be investigated. We will use a prospective, multimethods concurrent observational design comprising two studies. Study 1 comprises a suite of surveys administered to BrainTrack users at five time points and interviews with BrainTrack users. Study 2 involves interviews with GPs.

The following aims will be addressed:

### Study 1

1. To evaluate user experience and implementation outcomes (eg, help-seeking, uptake) of the BrainTrack app in the real-world context.
2. To evaluate whether app use is associated with improvements in users’ dementia literacy, dementia knowledge, perceptions of dementia-related stigma and help-seeking.
3. To determine the economic and social value of the BrainTrack app.

### Study 2

1. To elicit perceptions from GPs about the usefulness of the BrainTrack app and the app Insight Feedback Report in facilitating discussions with patients about cognition and dementia.

## Methods and analysis

### Study 1

#### Design

For Study 1 a multimethods observational design will be used, comprising a suite of surveys administered to BrainTrack users at five time points and semi-structured telephone interviews with a subset of users about their perceptions and experience of BrainTrack. This study did not use patient co-design during protocol development.

#### Target population

Australians aged over 50 years and without a diagnosis of dementia, mild cognitive impairment or stroke who download the BrainTrack app from October 2022 to May 2023 will be invited to participate in the study at the time they download the app. A subset comprising 80–120 of these participants will be invited to take part in a semi-structured telephone interview about their perceptions of and experience with BrainTrack. At least 80 individuals taking part in the interviews will be current users of the BrainTrack app, and 20 individuals will have indicated on a recent survey that they ceased using BrainTrack. Interview participation will be invited based on four criteria: gender, location (metropolitan, regional or rural), if English is the primary language spoken at home (yes/no) and Aboriginal and/or Torres Strait Islander (yes/no), with the aim of recruiting a minimum of five users in each stratum.

#### Setting

Recruitment will occur via the BrainTrack app. Data collection will occur online (surveys, Google Analytics, Dementia Australia Helpline data) and via teleconferencing (interviews).

#### Survey measures

Survey data will be collected using Research Electronic Data Capture (REDCap) hosted by Deakin University. A total of five online study surveys will be completed over 12 months. Refer to table 1 for a full list of outcomes, data sources and data collection time points.

**Table 1:** Study outcomes, measures and data collection time points

Dimension/outcomes	Measure/tool	When
Subjective memory concern	Subjective Memory Complaint Questionnaire – 14 item, validated	Baseline
User experience	User Experience Questionnaire	1, 3, 6, 12 months
Acceptability/satisfaction	Open-ended survey questions (eg, What did you like about using the app?)Interview	1, 3, 6, 12 months6+months
Adoption (intention to seek advice if needed)	Single survey item (eg, Do you intend to seek advice from your GP or the Dementia Australia Helpline about your brain health?)Interview	1, 3, 6, 12 months6+months
Appropriateness/usefulness	Single survey item (eg, Would you recommend BrainTrack to others?)Interview	1, 3, 6, 12 months6 months
Feasibility	Single survey item (eg, Were there things that made it easy to use BrainTrack?)Interview	1, 3, 6, 12 months6+months
Barriers and facilitators	Open ended survey questions (eg, Have you experienced any barriers to using BrainTrack?)Interview	1, 3, 6, 12 months6+months
Dementia literacy	Consumer Access, Appraisal and Application of Services and Information for Dementia Scale	Baseline, 1, 3, 6, 12 months
Dementia knowledge	Dementia Knowledge Assessment Scale	Baseline, 1, 3, 6, 12 months
Stigma	Dementia Public Stigma Scale	Baseline, 1, 3, 6, 12 months
Motivation to change	Motivation to Change Lifestyle and Health Behaviours for Dementia Risk Reduction Scale	Baseline, 1, 3, 6, 12 months
Help-seeking	Survey single item: Did you contact your GP and/or Helpline about memory concerns?Dementia Australia Helpline data	6, 12 monthsWeekly, for 12 months
Adoption/uptake and penetration/spread	Google Analytics # Destinations played # Games played within each destination # Rounds played within each game	Each time app is used
Utilisation (time spent in the app and what elements they navigate)	Google Analytics Event-coded, time stamp data Elements navigated (games, education information/resources, insight report) – navigation graph constructions	Each time app is used
Fidelity/adherence and sustainability	Google Analytics Login dates Monthly use for 12 months	Each time app is used
Cognitive performance	App performance (accuracy) score for each round and overall game	Each time app is used

GP, general practitioner.

Demographic data will include postcode, gender (male, female, other, rather not say), year of birth, primary language spoken at home, Aboriginal and/or Torres Strait Islander origin (yes/no), education level (primary school only, high school (not completed), high school (completed), diploma, bachelor’s degree, postgraduate), concern about memory or thinking (yes/no), noticed change in memory or thinking over the past 12 months (yes/no), current diagnoses (dementia, mild cognitive impairment, stroke, diabetes), how users learnt about the app (Dementia Australia website, social media, referred to by a family member, referred to by a friend, other) and reason/s for using the app (concern about my brain health, I have a family history of dementia, I want to keep mentally active, I like playing digital games, other).

The Subjective Memory Complaints Questionnaire (SMCQ),^51^ which consists of 14 dichotomous (yes/no) response items, reflects metacognition of general and specific memories. The scale yields three scores: total score, global memory function and everyday memory function, with higher SMCQ scores indicative of a more severe subjective memory problem. The SMCQ has been found to have adequate reliability (α=0.86) and has been shown to discriminate well between the elderly with and without dementia (p<0.01).^51^

The Consumer Access, Appraisal and Application of Services and Information for Dementia (CAAASI-Dem)^52^ will examine individuals’ self-assessed confidence in their ability to access, appraise and use dementia services and information. It is a 26-item measure using either a 5-point Likert scale (eg, not at all confident—extremely confident) with five factors: evaluation and engagement, readiness, social supports, specific dementia services and practical aspects. CAAASI-Dem has demonstrated adequate convergent and divergent validity, and the subscales have been shown to have very good internal reliability (α=between 0.85 and 0.94).^53^

The Dementia Public Stigma Scale (DePSS)^54^ will measure perceived dementia-related public stigma among community-dwelling adults. It is a 16-item, 7-point Likert scale (1=strongly disagree, 7=strongly agree) instrument consisting of five factors: fear and discomfort, negative perceptions, positive perceptions, burden and exclusion. The DePSS has been shown to have adequate internal reliability (α=between 0.74 and 0.81).^55^

The Dementia Knowledge Assessment Scale (DKAS)^56^ is a 25-item scale using a 5-point Likert-type scale where 1=true and 4=false with an additional option (5=do not know) to measure dementia knowledge characteristics. It produces four consistent domains: causes and characteristics, communication and behaviour, care considerations and risks and health promotion. The DKAS has shown good discriminant validity and acceptable internal reliability of subscales (α=between 0.65 and 0.76).^56^

The Motivation to Change Lifestyle and Health Behaviours for Dementia Risk Reduction Scale (MCLHB-DRR)^57^ measures beliefs underpinning the lifestyle and health behavioural changes needed for dementia risk reduction. The MCLHB-DRR consists of 27 items using a 5-point Likert-type scale ranging from 1 (strongly disagree) to 5 (strongly agree). It contains seven subscales of the Health Belief Model: perceived susceptibility, perceived severity, perceived benefits, perceived barriers, cues to action, general health motivation and self-efficacy. The measure has demonstrated adequate internal and test-retest reliability (α=between 0.61 and 0.86 and α=between 0.55 and 0.78, respectively) in participants aged >50 years.^57^

The User Experience Questionnaire (UEQ)^58^ will measure app usability and contains 26 semantic differential items (with opposing descriptive words at each end, eg attractive—unattractive), measured on a 7-point scale. The UEQ measures usability and quality of user experience and has demonstrated acceptable internal reliability (α=between 0.69 and 0.88).^59^

Investigator-developed items/open-ended questions will be used to elicit perceptions of implementation (ie, acceptability/satisfaction, adoption (intention to seek advice), appropriateness/usefulness, feasibility, barriers to and facilitators of app use). The surveys will also ask participants about help-seeking (ie, whether they contacted the Dementia Helpline and/or GP).

##### App usage and Google Analytics data

We will track how users navigate through the app pages and fields, how long they dwell on a page and how often they return to the app. Google Analytics data will capture adoption/uptake and penetration/spread, utilisation (navigation and dwell time on games and education information/resources), fidelity/adherence and sustainability (monthly use for 12 months).

### Interviews

Semi-structured interviews will tap relevant influences on BrainTrack users’ behaviour, their actual or intended help-seeking behaviour and preferences, perceptions and experiences of the app (online supplemental file 2, Interview Guides). Interviews will be conducted by telephone or a proprietary videoconferencing software programme (eg, Microsoft Teams), and audio-recorded and transcribed verbatim. Participants will be offered a $A25 gift voucher to acknowledge their time to complete the interview.

### Dementia Australia Helpline call data

Helpline operators routinely collect standard information (postcode, age, gender, the reason for the call, whether the person has a dementia diagnosis, cultural background, Indigenous background, need for an interpreter). From October 2022, they will also record whether the call was triggered by the BrainTrack app. Dementia Australia will produce weekly aggregated data on the proportion of Helpline calls triggered by BrainTrack use across a 12-month period by demographic characteristics (eg, age, gender, most frequently used language and/or Indigenous background).

### Procedures

People downloading the BrainTrack app will complete a screening questionnaire; those eligible will be invited to participate in the study. Users who wish to take part will be contacted via a personalised email link to provide consent to participate, then complete a baseline survey and subsequent follow-up surveys at 1, 3, 6 and 12 months from baseline. Surveys comprise a suite of brief instruments and are anticipated to take approximately 20 min to complete. Consent to participate also involves approval for the collection and use of Google Analytics app usage data and monthly BrainTrack activity data. Once they have been in the study for 6 months, a subset of the participants will be invited to take part in a semi-structured telephone interview about their experiences and perceptions of BrainTrack. To acknowledge the time commitment to complete the surveys, a $A50 gift voucher will be offered to participants following completion of all five surveys.

### Survey data analysis

Aim 1: to evaluate the user experience and implementation outcomes across measurement times. Outcomes will be summarised at each measurement time (longitudinally) overall and across different subgroups defined by key socio-demographic characteristics such as socio-economic status,^60^ place of residence (rural/urban), gender, age group, Aboriginal and/or Torres Strait Islander status and level of education. The longitudinal pattern of each outcome of interest across subgroups will be compared using linear mixed models (LMM), with participants as a random effect to account for repeated measures and adjusting for potential confounders of the association between outcome and the socio-demographic factor.

Aim 2: to evaluate whether app use is associated with improvements in users’ dementia literacy, dementia knowledge, perceptions of dementia-related stigma and help-seeking. From Google Analytics data, cumulative measures of use of the educational component of the app between baseline and each survey time, for all participants, will be derived. LMMs will estimate the association between each outcome (dementia literacy, dementia knowledge and stigma scores and help-seeking from survey data) and usage score adjusted for baseline characteristics. Missing data. Drop-out is likely to be driven by the outcomes of interest (ie, user experience, usability). We will collect reasons for withdrawal to inform the missing data approach, that is, multiple imputation or inverse probability of censoring weighting.

Aim 3: to determine the economic and social value of the BrainTrack app.

SROI analysis,^61 62^ a form of cost-benefit analysis, will be conducted to evaluate the economic and social value generated by the BrainTrack app. Outcomes collected via the surveys and open-ended questions (at 1, 3, 6 and 12 months) will measure the extent to which the app influenced each outcome. Outcomes will be monetised using financial proxies obtained from published literature and existing social value banks. The monetised outcomes will be discounted on the basis of what would have happened without using the app (deadweight), what outcomes are displaced by the app (displacement), who else has contributed to the outcomes aside from the funder (attribution), and whether experience of the outcomes declines over time (drop off), based on responses to the rating-scale questions. Finally, the discounted, monetised value of outcomes will be divided by total investment (inputs) to estimate the SROI ratio; where the value of the benefits is greater than the costs, that is, an SROI ratio >1, then the investment is worthwhile.

### Google Analytics data analysis

To understand app user behaviour, Google Analytics data will be summarised as number of interactions (eg, tutorial completion, app removed, session start and restart), number of users by app destination (ie, Australia, Indonesia, China), number of users by app version and number of error logs. Stochastic navigation graphs will indicate the preferred content and navigation pathways and, when combined with dwell time data, will enable a model that provides specific guidance on aspects of the app that work and where further modifications are needed. Aggregated data from non-study participants will give insight into usage patterns and the representativeness of the study sample compared with the entire app user population.

### Interview data analysis

Interview data will first be inductively analysed using a thematic analysis technique to identify patterns within the data. We will follow the six-step process proposed by Braun and Clarke:^63^ familiarisation, coding, generating themes, reviewing themes, defining and naming themes and writing up. Next, the Theoretical Domains Framework (TDF)^64^ domains and constructs will provide a coding schema to guide deductive analysis of the interview data. The domains are derived from 33 theories relevant to implementation efforts. The TDF (V.2)^64^ comprises 14 domains of influence on behaviour across cognitive, affective, social and environmental areas. The findings will be mapped using the Theory and Techniques Tool^65^ to identify suitable evidence-based behaviour change techniques. The tool provides a matrix, including the domains of the TDF and 74 behaviour change techniques. Interview data will be managed using NVivo software.

### Power considerations

The overall target sample size (n=1000) is large enough to produce accurate estimates of the mean value of the outcomes of interest at each time point. This sample will also allow the identification of key demographic factors associated with each outcome. Conservatively, assuming a prevalence of 10% for a binary exposure factor, for example, rural/urban location, 1000 participants (or 500 participants under a very large attrition rate) will provide 90% power (two-sided test, α=0.05) to detect a small/moderate standardised effect (Cohen’s d) of 0.34 (n=1000) or 0.48 (n=500) between the two exposure groups at one measurement time. Smaller effects will be detectable for exposure factors with a higher prevalence. For factors with three levels (eg, education or age groups) with approximately 333 participants (166) in each subgroup, we will have 90% power to detect a standardised effect of 0.29 (0.41) (two-sided test, α=0.015 to account for multiple comparisons) in at least one of the three pairwise comparisons. Power analysis was conducted using PASS Software (V.2021).

### Study 2

#### Design

For Study 2 a qualitative design will be used, involving semi-structured interviews with GPs about the perceived usefulness of the BrainTrack app and the Insight Feedback Report.

#### Participants

GPs currently practicing in Australia will be invited, via physical and digital flyers distributed through The Royal Australian College of General Practitioners networks, to take part. Efforts will be made to recruit GPs from all Australian states and territories and include GPs of varying years of experience practising in rural and metropolitan settings. The final sample size will be determined by the point at which information redundancy is reached^66^; we expect this to be achieved after 20 interviews.^67^

#### Setting

Data collection will occur via teleconferencing (interviews).

#### Interviews

Semi-structured interviews will cover prior experience and/or knowledge of BrainTrack, perceived benefits and potential barriers to patient use of the app, general suggestions for improvement and perceptions of the usefulness of the Feedback Report in facilitating GP/patient discussions about cognition and dementia (online supplemental file 2, Interview Guides). GPs will be asked to indicate their gender, age, postcode of their practice, an estimate of the percentage of their patients that are culturally and linguistically diverse and the number of years they have been practising as a GP. A full list of interview questions verbal consent will be obtained before commencing interviews, which will be conducted by telephone or a proprietary videoconferencing software programme (eg, Microsoft Teams), and audio-recorded and transcribed verbatim. Participants will be offered a $A200 gift voucher to acknowledge their time to complete the interview.

#### Qualitative analyses

Analysis of GP interview data will follow the procedure for interview data analysis outlined for Study 1.

### Data management (Studies 1 and 2)

#### Collection, storage and transfer of data

Data will be collected and stored entirely electronically. Quantitative data will be collected using REDCap, a secure web-based survey hosted on the Deakin University server. REDCap will be accessed by consenting app participants from the app landing page and personalised survey links. On completion and closure of the survey, data will be exported from REDCap without identifiers for cleaning and analysis. The Google Analytics data are automatically collected from app users and will be accessed by the members of the research team who developed the app. The semi-structured interview audio recordings will be collected by telephone or using an online videoconferencing platform such as Microsoft Teams, enabling the security of the recording. At the completion of each interview, the audio recording will be transferred to the secure server and deleted from the videoconferencing platform. The interviews will be transcribed by a professional transcription service into Microsoft Word files. These will be imported into NVivo data analysis software. Study data will only be accessible to members of the research team who require access for analysis purposes and have ethics approval to access the data.

#### Preservation and future use

Deakin University will hold exclusive ownership of the data, which will be retained for 7 years from the completion of the final publication of this research. If the data are to be used in future research projects, it will only be accessible in de-identified form, subject to further ethical approval.

#### Study status and timeline

The study will be open for participant recruitment for 6 months after the release of the BrainTrack app, and data collection will occur for 12 months after the enrolment of the last participant. The study commenced in October 2022, coinciding with the launch of BrainTrack by Dementia Australia. Refer to online supplemental figure S1 for a detailed study timeline.

### Patient and public involvement

Two consumer advocates, who bring lived experience of dementia and one of whom was involved in the initial conception of the app, will advise on the conduct of the project from a consumer perspective. This will involve participation in steering committee meetings and contributing to the interpretation and dissemination of findings.

A summary of results arising from this study will be made available to all participants via email at the conclusion of the study. Participants will be informed of this and invited to register their interest via the plain language statements.

## Ethics and dissemination

This study has received Human Research Ethics Committee approval from the Deakin University Human Research Ethics Committee (Study 1: HREC Reference Number 2022–220) and Deakin University Human Ethics Advisory Group, Faculty of Health (Study 2: Reference Number 202_2022) and will be carried out in line with the Australian Code for the Responsible Conduct of Research.^68^ Any modification to the study as described here will be subject to further ethical review prior to implementation. Informed consent will be obtained prior to participation and will be obtained verbally for interviews and online for surveys. Study findings will be published in peer-reviewed journals and communicated to key stakeholders.

## Discussion

Using a prospective multimethods concurrent two-study observational design, we will evaluate the user experience and implementation outcomes of BrainTrack, a unique and innovative app designed to promote brain health self-awareness, self-determination to promote help-seeking and, ultimately, a timelier dementia diagnosis. BrainTrack is informed by theory, research and co-design and is freely available to all members of the community.

This study has some limitations. There are several possible sources of bias and confounding factors in this study design. First, survey drop-out is likely to be driven by the outcomes of interest (ie, user experience, usability). To account for this, we will seek to interview a sample of people who cease use of BrainTrack, to gain an understanding of their experience and if it influenced their decision to discontinue use. Reasons for withdrawal from the study will be obtained, and patterns in missing data will be examined to inform our approach to the treatment of the missing data, that is, multiple imputation or inverse probability of censoring weighting.

The app is available in English only and there may also be a group of older Australians who still do not have access to a smartphone or tablet or do not know how to use these devices. Thus, it is possible that there will be bias in the sample against linguistically diverse populations or those with limited access to digital devices and will be acknowledged as limitations. Nevertheless, this study could form the blueprint for other languages and countries to adopt versions of the app in future. Furthermore, the app is a tremendously scalable tool with a reach comparable to other currently available apps and an increasing proportion of the population will have access over time to smart devices with the increasing rates of smartphone uptake and tech savviness.

A limitation of interviews is that individuals consenting to participate may do so because they have strong views in favour of or against the app or are enticed by the prospect of financial reimbursement. Hence, a bias in responses is possible and will be acknowledged as a limitation. As noted above, we will interview participants who cease use of the app to understand their reasons for cessation and the level of compensation offered will be proportionate to the time commitment required for participation, approved by an independent ethics committee and below an amount that could be considered an incentive.

This study has a number of strengths. A multi-inducement methods approach will be used to collect data from a wide range of sources, enabling a comprehensive understanding of user experience and implementation outcomes from a range of perspectives. Qualitative and quantitative data will result in more robust findings than would be possible with a single methodology. Capturing the perspectives of BrainTrack users who continue and those who cease use, as well as the perceptions of GPs, will provide a comprehensive understanding of user experience, implementation outcomes and the influence of BrainTrack on a range of factors that are important in determining help-seeking (including perceived dementia-related stigma, dementia literacy and knowledge) and ultimately a timely dementia diagnosis.

The findings of this research will inform future iterations and improvements to the BrainTrack app design and content, further understanding of the reach and uptake of the app over time to inform ongoing implementation and scale-up, as well as inform strategy and resourcing for Dementia Australia Helpline consultants to complement and support future use of the app. The findings will also inform a strategy to promote BrainTrack to GPs. Additionally, the findings will inform the development of resources to complement BrainTrack for use by the community, GPs and other health professionals. This study will also provide a blueprint for future digital technology translation and evaluations and a foundation to promote widespread use of BrainTrack to help facilitate timely dementia diagnosis.

## Supplementary material

10.1136/bmjopen-2024-088182 online supplemental file 1

10.1136/bmjopen-2024-088182 online supplemental file 2
