# Supplementary material for: Implementing innovative technology promoting self-awareness of brain health and self-determination in obtaining a timely dementia diagnosis: protocol for a multimethods, concurrent, two-part observational study
Source: BMJ Open. 2025 Jun 17;15(6):e088182. doi: 10.1136/bmjopen-2024-088182 (PMC12182042; doi:10.1136/bmjopen-2024-088182)
Supplement: online supplemental file 1 [file bmjopen-15-6-s001.docx]

**S1 Fig. Study Gantt chart.**

|  | **PROJECT ACTIVITIES** | **MILESTONE** | **2022** | | **2023** | | | | **2024** | | | | | **2025** | |
| --- | --- | --- | --- | --- | --- | --- | --- | --- | --- | --- | --- | --- | --- | --- | --- |
|  |  |  | Q3 | Q4 | Q1 | Q2 | Q3 | Q4 | Q1 | Q2 | Q3 | Q4 | | Q1 | Q2 |
|  | Administration | Contracts finalised, appointment of staff |  |  |  |  |  |  |  |  |  |  | |  |  |
|  | Research Ethics Approval | Deakin University |  |  |  |  |  |  |  |  |  |  | |  |  |
| Surveys | Recruitment & data collection | Recruitment & baseline data |  |  |  |  |  |  |  |  |  |  | |  |  |
|  |  | 1-month surveys |  |  |  |  |  |  |  |  |  |  | |  |  |
|  |  | 3-month surveys |  |  |  |  |  |  |  |  |  | |  |  |  |
|  |  | 6-month surveys |  |  |  |  |  |  |  |  |  |  | |  |  |
|  | Data collection | 12-month surveys |  |  |  |  |  |  |  |  |  |  | |  |  |
|  |  | Google analytics |  |  |  |  |  |  |  |  |  |  | |  |  |
| Interviews | BrainTrack users & ceased users | Recruitment and interviews |  |  |  |  |  |  |  |  |  |  | |  |  |
|  |  | Interview transcription |  |  |  |  |  |  |  |  |  |  | |  |  |
|  | General Practitioners | Explainer video production |  |  |  |  |  |  |  |  |  |  | |  |  |
|  |  | Recruitment and interviews |  |  |  |  |  |  |  |  |  |  | |  |  |
|  |  | Interview transcription |  |  |  |  |  |  |  |  |  |  | |  |  |
| Analysis | Data analysis | Processing of quantitative data |  |  |  |  |  |  |  |  |  |  | |  |  |
|  |  | Quantitative analysis BrainTrack users |  |  |  |  |  |  |  |  |  |  | |  |  |
|  |  | Qualitative analysis BrainTrack users & GPs |  |  |  |  |  |  |  |  |  |  | |  |  |
| Reporting | Project management | Steering Committee Meetings |  |  |  |  |  |  |  |  |  |  | |  |  |
|  | Reporting & preparation of manuscripts | MRFF/Ethics annual & final reports |  |  |  |  |  |  |  |  |  |  | |  |  |
|  |  | Manuscripts produced |  |  |  |  |  |  |  |  |  |  | |  |  |
|  | Final Reports | Deliverables & recommendations |  |  |  |  |  |  |  |  |  |  | |  |  |
