# Supplementary material for: Implementing innovative technology promoting self-awareness of brain health and self-determination in obtaining a timely dementia diagnosis: protocol for a multimethods, concurrent, two-part observational study
Source: BMJ Open. 2025 Jun 17;15(6):e088182. doi: 10.1136/bmjopen-2024-088182 (PMC12182042; doi:10.1136/bmjopen-2024-088182)
Supplement: online supplemental file 2 [file bmjopen-15-6-s002.docx]

**S2 Interview Guides (App users, Ceased app users and GPs).**

Guide for interview with app users

*Preamble*

Thank you for agreeing to participate in an interview. Based on your reading of the participant information and consent form, do you have any questions about this research project?

As a reminder, I will be recording this interview and a professional company bound to confidentiality will transcribe the audio-recording. Is this ok?

The data obtained from this interview will be analysed along with other interview data and the findings of this study and will likely be published in peer-reviewed journals and presented at conferences. We can send you a copy of the final report on request.

I will ask you a series of questions with some prompts. If there are any questions you would prefer not to answer, please say so – there is no need to explain why you don’t wish to answer.

As a reminder, this research study is about your use of the app. We are interested to know about your experiences and perspectives of using *BrainTrack*. We are also interested in how you think *BrainTrack* can be improved for future use.

Before I ask the first interview question, do you have any other questions for me?

OK. Are you happy for me to start recording now?

*Once recording has commenced* *ask* - I have started recording our conversation, and everything discussed from this point forward will be transcribed into a record of the interview. For the transcribed record, could you please confirm that you are providing your consent to participate in this interview?

__________________________________________________________________________________

Questions:

***User experience***

How often do you use BrainTrack?

On what device are you using BrainTrack e.g., a tablet or smart phone?

Do you think using a different device would make it easier to use BrainTrack? E.g., a smart phone, tablet, laptop, desktop computer?

Do you find *BrainTrack* easy to see on the screen?

Did you complete all the games?

- Yes: were they enjoyable? and what did you like about them?
- No: why not? How do you think they could be improved?

Do you find it easy to use BrainTrack?

- If so, can you tell me more what makes it easy for you to use BrainTrack?

- If not, can you tell me more about what made it challenging?

Do you think BrainTrack could be made easier to use?

If so, how do you think this could be done?

What did you like about using *BrainTrack*?

What did you NOT like about using *BrainTrack*?

***Actual or intended help-seeking behaviour***

Have you looked at any resources linked to *BrainTrack*?

Has *BrainTrack* prompted you to call the Dementia Australia Helpline?

Do you keep track of your overall progress on *BrainTrack?* (Behavioural regulation)

Have you generated a copy of the report in BrainTrack?

Have you taken the report generated from *BrainTrack* to your GP?

If no, do you intend to take the report generated from *BrainTrack* to your GP?

If yes, would you be willing to provide their contact details so that we can ask them about their experience using the report?

Has *BrainTrack* prompted you to increase healthy behaviours? (e.g., eating healthy, exercising more, getting enough sleep, reducing alcohol intake).

***Perceived barriers and facilitators***

**Barriers**

Did you experience any challenges using BrainTrack?

- If yes, can you tell me about those challenges?

Do you find that you forget to use *BrainTrack?* (Memory, attention, and decision processes)

Do you find that you are easily distracted when using *BrainTrack?* (Memory, attention, and decision processes)

Do you get bored when using BrainTrack?

**Facilitators**

Do you believe you have the skills to use a digital device? E.g. phone or tablet (Skills) – related to aptitude with hardware

Do you believe you have the skills to play digital games? E.g. BrainTrack (Skills) – related to aptitude with software

Are you confident that you would be able to navigate any technical difficulties that may arise in *BrainTrack?* (Beliefs about capabilities)

Have you learned more about dementia from *BrainTrack?* (Knowledge)

Do you believe that using *BrainTrack* will improve your brain health? (Beliefs about consequences)

What other outcomes might there be from using *BrainTrack?* (Beliefs about consequences)

Do you intend to keep using *BrainTrack?* (Intention)

- If yes, do have a clear plan to regularly use *BrainTrack* e.g., every time you’re on the train, every day/week after dinner)? (Goals)
- If no, can you tell me why not?

Do any friends or family encourage you to use *BrainTrack?* (Social influences)

Are you aware of any evidence that links playing games to good brain health? (Knowledge)

- If yes, what are your thoughts about this evidence? (prompt: do you agree? etc.)

Do you feel as though you have enough knowledge about brain health? (knowledge)

- If yes, where did you go to get your information?
- If no, what would you like to know more about?
  - Where would you go to get this information?

**Other/finishing up**

Is there anything else you would like to add about your use of *BrainTrack?*

Would you like to review the transcript of this interview?

Would you like to receive a report of the findings from this study?

***Thank you for your time in providing valuable feedback about BrainTrack.***

Guide for interview with ceased app users

*Preamble*

Thank you for agreeing to participate in an interview. Based on your reading of the participant information and consent form, do you have any questions about this research project?

As a reminder, I will be recording this interview and a professional company bound to confidentiality will transcribe the audio-recording. Is this ok?

The data obtained from this interview will be analysed along with other interview data and the findings of this study and will likely be published in peer-reviewed journals and presented at conferences. We can send you a copy of the final report on request.

I will ask you a series of questions with some prompts. If there are any questions you would prefer not to answer, please say so – there is no need to explain why you don’t wish to answer.

As a reminder, this research study is about your use of the app. We are interested to know about your experiences and perspectives of using *BrainTrack*. We are also interested in how you think *BrainTrack* can be improved for future use.

Before I ask the first interview question, do you have any other questions for me?

OK. Are you happy for me to start recording now?

*Once recording has commenced* *ask* - I have started recording our conversation, and everything discussed from this point forward will be transcribed into a record of the interview. For the transcribed record, could you please confirm that you are providing your consent to participate in this interview?

__________________________________________________________________________________

Questions:

***Reasons for stopping***

I think you have indicated that you have stopped using BrainTrack, is that correct?

Do you remember when you last used the app?

Can you tell me about what influenced your decision to stop using BrainTrack?

Can you tell me about any challenges you experienced when you were using BrainTrack?

What was it about *BrainTrack* that drew you to use it initially?

Do you think the app fulfilled your initial expectations? How / why not?

***User experience***

These questions are about when you were using BrainTrack…

How often did you use BrainTrack?

On what device were you using BrainTrack e.g., a tablet or smart phone?

Do you think using a different device would have made it easier to use BrainTrack? E.g., a smart phone, tablet, laptop, desktop computer?

Did you find *BrainTrack* easy to see on the screen?

Did you complete all the activities at each travel destination?

- Yes: were they enjoyable? and what did you like about them?
- No: why not? How do you think they could be improved?

Thinking about the app in general, did you find it easy to navigate the BrainTrack app?

- If so, can you tell me more what makes it easy for you to use BrainTrack?

- If not, can you tell me more about what made it challenging?

Do you think BrainTrack could be made easier to use?

If so, how do you think this could be done?

***Actual or intended help-seeking behaviour***

Have you looked at any resources linked to *BrainTrack*?

Has *BrainTrack* prompted you to call the Dementia Australia Helpline?

Did you keep track of your overall progress on *BrainTrack?* (Behavioural regulation)

Have you generated a copy of the report in BrainTrack?

Have you taken the report generated from *BrainTrack* to your GP?

If no, do you intend to take the report generated from *BrainTrack* to your GP?

If yes, would you be willing to provide their contact details so that we can ask them about their experience using the report?

Has *BrainTrack* prompted you to increase healthy behaviours? (e.g., eating healthy, exercising more, getting enough sleep, reducing alcohol intake).

***Perceived barriers and facilitators***

**Barriers**

Do you find that you forgot to use *BrainTrack?* (Memory, attention, and decision processes)

Do you find that you were easily distracted when using *BrainTrack?* (Memory, attention, and decision processes)

Did you get bored when using BrainTrack?

**Facilitators**

Do you believe you have the skills to use a digital device? E.g. phone or tablet (Skills) – related to aptitude with hardware

Do you believe you have the skills to play digital games? E.g. BrainTrack (Skills) – related to aptitude with software

Are you confident that you would be able to navigate any technical difficulties that may arise in *BrainTrack?* (Beliefs about capabilities)

Have you learned more about dementia from *BrainTrack?* (Knowledge)

Do you believe that using *BrainTrack* will improve your brain health? (Beliefs about consequences)

What other outcomes might there be from using *BrainTrack?* (Beliefs about consequences)

Do you intend to use *BrainTrack* again in the future*?* (Intention)

- If yes, do have a clear plan to regularly use *BrainTrack* e.g., every time you’re on the train, every day/week after dinner)? (Goals)
- If no, can you tell me why not?

Did any friends or family encourage you to use *BrainTrack?* (Social influences)

Are you aware of any evidence that links playing games to good brain health? (Knowledge)

- If yes, what are your thoughts about this evidence? (prompt: do you agree? etc.)

Do you feel as though you have enough knowledge about brain health? (knowledge)

- If yes, where did you go to get your information?
- If no, what would you like to know more about?
  - Where would you go to get this information?

**Other/finishing up**

Is there anything else you would like to add about your use of *BrainTrack?*

Would you like to review the transcript of this interview?

Would you like to receive a report of the findings from this study?

***Thank you for your time in providing valuable feedback about BrainTrack.***

Guide for interview with GPs

*Preamble*

Thank you for agreeing to participate in an interview. Based on your reading of the participant information and consent form, do you have any questions about this research project?

As a reminder, I will be recording this interview and a professional company bound to confidentiality will transcribe the audio-recording. Is this ok?

The data obtained from this interview will be analysed along with other interview data and the findings of this study and will likely be published in peer-reviewed journals and presented at conferences. We can send you a copy of the final report on request.

I will ask you a series of questions with some prompts. If there are any questions you would prefer not to answer, please say so – there is no need to explain why you do not wish to answer.

Before I ask the first interview question, do you have any other questions for me?

Are you happy for me to start recording?

*Once recording has commenced* *ask* - I have started recording our conversation, and everything discussed from this point forward will be transcribed into a record of the interview. For the transcribed record, could you please confirm that you are providing your consent to participate in this interview?

__________________________________________________________________________________

Demographics:

What is your gender?

What is your age?

What is the postcode of your practice?

What percentage of your patients are culturally and linguistically diverse?

What is your role in healthcare?

For how many years have you been a GP / health professional?

Questions:

Had you heard of BrainTrack prior to this study?

If yes: Where did you hear about it?

Have any of your patients reported using it?

Have any of your patients discussed their *BrainTrack* report with you?

***Perceived barriers and facilitators***

**Barriers**

Do you find it difficult to understand the Insight feedback results report generated by *BrainTrack*? (Beliefs about Capabilities)

If so, what were the challenges?

Would you find it hard to initiate a conversation about brain health with a patient who was using *BrainTrack*?

If so, why?

What do you dislike about *BrainTrack*?

**Facilitators**

Do you feel you have enough information about *BrainTrack* to facilitate discussions with patients about dementia and the diagnostic pathway?

Would you recommend *BrainTrack* to your patients? Why? Why not?

Have you learned more about dementia from *BrainTrack?* (Knowledge)

Do you believe that using *BrainTrack* will lead to earlier dementia diagnosis? (Beliefs about consequences)

What other benefits might there be from using *BrainTrack?* (Beliefs about consequences)

Do you have a strong intention to encourage your patients to use *BrainTrack?* (Intention)

Are you aware of the resources through Dementia Australia or your local health pathways which would help you to support your patients who are using *BrainTrack*? (Knowledge)

- If yes, what are your thoughts about these resources? (prompt: do you agree? etc.)

*Questions about insight feedback report*

What do you like about *BrainTrack* and the Insight feedback report?

How much time would you allow to explore the report further?

What would you say to a patient if you were presented with this report?

What would be your next step if you were concerned about a patient's cognitive function based on the Insight feedback report?

Would you recommend the app to patients with chronic conditions or multiple co-morbidities that increased their risk of dementia?

Do you feel you have sufficient resources to implement dementia risk reduction strategies with your patients who are using *BrainTrack*?"

***Areas for improvement***

Do you think the usability of *BrainTrack* and the Insight feedback report could be improved? If so, how?

Do you think there are other resources needed to support the use of BrainTrack

**Other/finishing up**

Is there anything else you would like to add about your use of *BrainTrack?*

Would you like to review the transcript of the recording of this interview?

Would you like to receive a report of the findings from this study?

***Thank you for your time in providing valuable feedback for this study.***
