# Supplementary material for: Implementing innovative technology promoting self-awareness of brain health and self-determination in obtaining a timely dementia diagnosis: protocol for a multimethods, concurrent, two-part observational study
Source: BMJ Open. 2025 Jun 17;15(6):e088182. doi: 10.1136/bmjopen-2024-088182 (PMC12182042; doi:10.1136/bmjopen-2024-088182)
Supplement: Supplementary file 3 [file bmjopen-2024-088182.reviewer_comments.pdf]

PEER REVIEW HISTORY

BMJ Open publishes all reviews undertaken for accepted manuscripts. Reviewers are asked to complete a checklist review form and are provided with free text boxes to elaborate on their assessment. These free text comments are reproduced below.

ARTICLE DETAILS

Title (Provisional)

Implementing innovative technology promoting self-awareness of brain health and self-determination in obtaining a timely dementia diagnosis: protocol for a multi-methods, concurrent, two-part observational study

Authors

Hutchinson, Alison M.; Macpherson, Helen; Petrovich, Tanya; Vasa, Rajesh; Chong, Terence W. H.; Engel, Lidia; Botha, Tanita; Bucknall, Tracey K.; Burns, Kelly; Daly, Stephanie; Lomas, Justine; Mouzakis, Kon; Mowszowski, Loren; Naismith, Sharon L; Redley, Bernice; Rivera Villicana, Jessica; Vouliotis, Andrew; Yuen, Eva; Orellana, Liliana

---

VERSION 1 - REVIEW

**Reviewer**	**1**
**Name**	**Mueller, Notger**
**Affiliation**	**University of Potsdam**
**Date**	**07-May-2025**
**COI**	**None**

Overall, the planned study is of high interest to both scientists and clinicians working in the field of dementia. The study protocol is, however, very ambitious and I am worried about recruitment and drop-out given the large number of interactions with the participants. Now, here is my major concern: the authors state that the study starts in October 2022 and will last for one year. Hence, data collection should be completed by now and instead of presenting only the study protocol the authors could at least make statements about the success of their recruitment strategy. Otherwise, one has to ask what additional merit publication of a study protocol has when the study is already done and we can expect to see the results in near future. The other major concern is that I haven't found information on the registration of the trial, also not all CONSORT guidelines (e.g. flow chart) seem to have been met.

VERSION 1 - AUTHOR RESPONSE

Reviewer: 1

Dr. Notger Mueller, University of Potsdam

Comments to the Author:

Overall, the planned study is of high interest to both scientists and clinicians working in the field of dementia. The study protocol is, however, very ambitious and I am worried about recruitment and drop-out given the large number of interactions with the participants. Now, here is my major concern: the authors state that the study starts in October 2022 and will last for one year. Hence, data collection should be completed by now and instead of presenting only the study protocol the authors could at least make statements about the success of their recruitment strategy. Otherwise, one has to ask what additional merit publication of a study protocol has when the study is already done and we can expect to see the results in near future. [NOTE FROM THE EDITORS: Pending the author response to the editorial query on the status of the study (above), this comment should be rebutted - results should not be included in the protocol]

Thank you for taking the time to review our protocol paper.

As this manuscript describes a study protocol, it is not appropriate to report recruitment outcomes. In future publications reporting the findings of this study, recruitment outcomes will be described in detail and in line with the relevant reporting guidelines.

There are several reasons why publishing the protocol at this stage, without including recruitment numbers, is meritorious. Publishing the protocol prior to reporting study outcomes provides greater detail regarding the conduct of the study than other publications reporting the results can accommodate, and it serves as a reference for future publications, providing transparency regarding the overall conduct of the study.

The other major concern is that I haven't found information on the registration of the trial, also not all CONSORT guidelines (e.g. flow chart) seem to have been met.

Registration of observational trials is not mandatory, nevertheless, we are seeking to publish the study protocol with BMJ Open to ensure a clear record of the intended study procedures to provide transparency regarding the overall conduct of the study.

Given that this is an observational study rather than a clinical trial, reporting in line with CONSORT guidelines is not applicable. We did adhere to relevant reporting guidelines and prepared the protocol paper in line with STROBE reporting guidelines for observational studies and attached a checklist detailing how we had met each applicable item within this protocol paper.
